# P2RY13 Exacerbates Intestinal Inflammation by Damaging the Intestinal Mucosal Barrier via Activating IL-6/STAT3 Pathway

**DOI:** 10.7150/ijbs.74304

**Published:** 2022-08-01

**Authors:** Xiaohan Wu, Shuchun Wei, Meilin Chen, Jinting Li, Yuping Wei, Jixiang Zhang, Weiguo Dong

**Affiliations:** 1 Department of Gastroenterology, Renmin Hospital of Wuhan University, Wuhan, Hubei Province, China.; 2 Key Laboratory of Hubei Province for Digestive System Disease, Wuhan, Hubei Province, China.

**Keywords:** ulcerative colitis, P2RY13, intestinal mucosal barrier disruption, STAT3, IL-6, MRS2211

## Abstract

The pathogenesis of ulcerative colitis (UC) is unclear, while genetic factors have been confirmed to play an important role in its development. P2RY13 is a G protein-coupled receptor (GPCRs), which are involved in the pathogenesis of inflammation and immune disorders. According to GEO database analysis, we first observed that the expression of P2Y13 was increased in UC patients. Therefore, we sought to determine the role of P2Y13 in the development of colitis. Our data showed that P2RY13 was highly expressed in the inflamed intestinal tissues of UC patients. In mice, pharmacological antagonism of P2Y13 can significantly attenuate the intestinal mucosal barrier disruption. In LPS-induced NCM460 cell, knockdown or pharmacological inhibition of P2RY13 increased the expression of intestinal tight junction protein and reduced apoptosis. In addition, we found that the effect of P2Y13 on colitis is related to the activation of the IL-6/STAT3 pathway. Activation of P2Y13 increases IL-6 expression and promotes STAT3 phosphorylation and nuclear transport. Deletion of the STAT3 gene in the intestinal epithelial cells of mice significantly mitigated the exacerbation of colitis due to P2Y13 activation. Thus, P2Y13 can aggravate intestinal mucosal barrier destruction by activating the IL-6/STAT3 pathway. P2Y13 might be a potential drug target for UC.

## Introduction

Ulcerative colitis (UC) is a type of chronic inflammatory bowel disease (IBD) 1 with complex pathogenesis. At present, it is believed that the etiology of UC involves genetic and environmental factors, immune dysregulation, and intestinal flora changes 2. The incidence and prevalence rates of UC are rising globally, consequently increasing the global economic burden 3. Therefore, a deeper understanding of the pathogenesis of UC is needed to optimize its treatment and reduce the disease burden.

Purinergic receptors are divided into P1 and P2. P2 receptors are further classified into P2Y receptors (P2YRs) and P2X receptors (P2XRs) 4. During inflammation or infection, cells secrete endogenous nucleotides such as ATP, ADP, and UTP, often as danger-associated molecular patterns (DAMPs), to elicit pro-inflammatory responses via cell-surface P2 purinergic receptor activation 5. Purinergic signal transduction is related to many pathological conditions, such as fibrotic liver disease, chronic kidney disease, diabetes, inflammatory bowel diseases, asthma, neurodegenerative diseases, and cancers 6-11. P2Y1 activation via ADP aggravates IBD through ERK5-mediated activation of NLRP3 inflammasome 12. P2X7R is involved in the pathogenesis of IBD by mediating the NLRP3/Caspase-1 inflammasome and NF-κB pathways, and regulating the balance of Th17 and Treg cells 13. In intestinal samples of patients with IBD, mRNA levels of both P2Y2 and P2Y6 receptors were found to be increased during flare-ups 14. Studies increasingly support the role of P2 purinergic receptors in the pathogenesis of IBD.

P2RY13 is a G_i_ protein-coupled receptor encoded by 354 amino acids. It is highly sensitive to ADP but can be activated by ADP and ATP 15. Gene deletion or drug inhibition of P2RY13 can relieve asthma via inhibiting IL-33 and HMGB1 release 11. MRS2211, an antagonist of P2RY13, inhibits LPS-induced IL-6 production in KUP5 cells and has the potential to downregulate hepatic inflammation 16. Evidence reveals the pivotal role of P2RY13 in inflammation and immune dysregulation. However, the contribution of P2RY13 to the development of IBD remains unclear.

In this study, the Gene Expression Synthesis (GEO) dataset showed that P2RY13 was upregulated in the intestinal tissues of patients with UC. Furthermore, we found that MRS2211-induced inhibition of P2RY13 activity significantly alleviated dextran sulfate sodium (DSS)-induced colitis. Our study demonstrated that P2RY13 played a key role in mediating UC development through disruption of the intestinal epithelial barrier via IL-6/STAT3 pathway activation.

## Materials and Methods

### Human colon samples

Two groups of human specimens were got in Wuhan University People's Hospital (Hubei, China). One group was intestinal inflammatory tissue taken from UC patients (n=15) by endoscopic biopsy, which were used for Western blotting. The intestinal tissues of healthy people (n=15) undergoing intestinal endoscopy without any gastrointestinal diseases were selected as normal controls. The other group was paraffin-embedded intestinal tissue (n = 30 UC and n = 30 normal tissues) collected from the department of pathology, which were used for immunohistochemical (IHC). Corresponding clinical data were obtained from clinicopathology and recorded in Table 1. The informed consent of all participants is obtained. The Study was approved by the institutional review committee (approval number: WDRY2021-K025).

### Mice

Animal experiments were approved by the Animal Care and Use Committee of Renmin Hospital of Wuhan University (PR China; approval number: 20181001). STAT3-deficient mice in IEC (STAT3^△IEC^) were produced by crossing STAT3 ^fl/fl^ (fl/fl) mice with Villin-Cre mice (GemPharmetech CO., Ltd, Nanjing, China). C57BL/6J WT mice were obtained from GemPharmetech CO., Ltd.

### Induction of Colitis

To induce colitis, C57BL/6 mice were given drinking water supplemented with 2.5% or 4% DSS (MP Biomedicals) for 7 days. The body weight, fecal consistency, and crude blood in feces and anus were observed once a day. Mice were sacrificed on the eighth day of DSS treatment, and the whole colon was taken and the length was measured. The terminal colon was fixed with 4% paraformaldehyde for 24h and embedded in paraffin. After that, the tissue was cut in to 3 μm slices for histological staining (H&E). The remaining colon was frozen with liquid nitrogen for PCR or Western blot (WB) analysis. Histological score of colonic injury as previously described 12.

### RNA Extraction and Real-Time PCR

Total RNA was extracted using Trizol (Invitrogen, USA) reagent. And using PrimeScript™ RT reagent Kit with gDNA Eraser (Perfect Real Time) (TaKaRa, Shiga, Japan; Cat No. RR047A) to synthesize cDNA. Real-time quantitative PCR was performed on a CFX Connect (BIO-RAD, USA) using TB Green Premix Ex Taq (TaKaRa; Cat. No. RR820A). Glyceraldehyde 3-phosphate dehydrogenase (GAPDH) served as the internal reference gene.

### Cell Lines and Cell Transfection

The human normal epithelial cell line NCM460 was grown in Dulbecco's modified Eagle medium (DMEM) supplemented with 10% fetal bovine serum at 37°C and 5% CO2. The siRNA were purchased from Sangon Biotech (Shanghai, China) including the siRNA targeting the human P2RY13 gene (siP2RY13) and nontargeting siRNAs (control siRNAs). NCM460 were plated in 6-well plates overnight and then transfected with siRNAs using Lipofectamine 2000 (Invitrogen, USA) according to the supplier's instructions. The siRNA sequences were: negative control: sense 5′-UUCUCCGAACGUGUCACGUTT-3′, antisense 5′-ACGUGACACGUUCGGAGAATT-3′; siP2RY13-1 sense 5′-CCACAGUGAUGCAAGGCUUTT-3′, antisense 5′-AAGCCUUGCAUCACUGUGGTT-3′; siP2RY13-2 sense 5′-GGCUCUGUGGGUGUUUGUUTT-3′, antisense 5′-AACAAACACCCACAGAGCCTT-3′; siP2RY13-3 sense 5′-CCAUAUACUCACAGUCAAATT-3′, antisense 5′-UUUGACUGUGAGUAUAUGGTT-3′.

### Western Blotting

Total protein was extracted from tissues and cells using the lysis buffer (50 mM Tris-HCl pH 7.4,1% NP-40, 0.5% Na-deoxycholate, 0.1% SDS,150 mM NaCl, 2 mM EDTA, 50 Mm NaF) with protease inhibitor. Extract nuclear proteins from cultured cells using a Nuclear and Cytoplasmic Protein Extraction Kit (P0027, Beyotime) according to manufacturer's instructions. The protein was carried out by 10% sodium dodecyl sulfate polyacrylamide gel electrophoresis (SDS-PAGE) and then transferred to the PVDF membrane (Bio-Rad Laboratories, Hercules, CA, USA). The membrane was sealed with 5% skim milk powder for 2h and then incubated at 4° in TBST diluted primary antibody for 8-12h. The membranes were incubated with TBST diluted secondary antibodies at room temperature for 1h. ChemiDocTMXRS+ system (BIO-RAD, USA), was used to detect protein signal. The primary antibodies used in this study: Bax (#50599-2-Ig,proteintech), STAT3 (#124H6,CST), P-STAT3 (#D3A7,CST), P2RY13 (#APR-017,Alomone labs), ZO-1 (#ab276131, abcam), LaminB (#12987-1-AP, proteintech), β-actin (#66009-1-Ig, proteintech), occluding (#91131,CST), Bcl-2 (#26593-1-AP, proteintech) and GAPDH (#60004-1-Ig, proteintech).

### Immunohistochemistry and AB-PAS

Paraffin-embedded mouse intestinal tissue was cut into 3 μm slices. The sections were stained by immunohistochemistry, using an UltraSensitive™ SP (mouse/rabbit) IHC kit (Maxib, Fuzhou, China), according to the manufacturer's instructions. AB-PAS was performed using an AB-PAS staining kit (Solarbio.G1285, Solarbio) according to the manufacturer's instructions. For IHC,primary antibodies against the following targets were used: ZO-1 (**#**ab276131, abcam), MUC-2 (**#**ab272692, abcam), P2RY13 (**#**APR-017, Alomone labs), STAT3 (**#**124H6, CST).

### Immunofluorescence staining

The cells on the slides were washed with PBS for 3 times, and then fixed with 4% paraformaldehyde at room temperature for 20 min. Cells washed with PBS for 3 times, and then permeabilized with 0.2% Triton X-100 for 15 min. Cells washed with PBS for 3 times, sealed with 5%BSA for 1h, and then incubated with primary antibody for 4 °C overnight. Cells were washed with PBS for 3 times, and then incubated the secondary antibody at 37 °C for 1h. Cells were imaged with Leica TCS SP8 confocal laser scanning microscope (Leica Microsystems, Wetzlar, Germany) and Olympus FV1200 (Japan).

### Electron Microscopy

The Fresh mouse intestinal tissue was fixed in 2.5% glutaraldehyde (pH 7.4) for 2h and then fixed in 1% osmic acid at 4 °C for 2h after washed three times with 0.1M phosphate buffer (pH 7.2). Then the intestinal tissue was gradient dehydrated in a graded series of ethanol and then was embedded in Epon-Araldite resin for penetration and placed in a model for polymerization. Then the semi thin section was used for positioning, and the ultrathin section was made and collected for microstructure analysis. After the counterstaining of 3% uranyl acetate and 2.7% lead citrate, the ultrathin section was observed with an HT7800 transmission electron microscope.

### TUNEL

Paraffin-embedded mouse intestinal tissue was cut in to 3 μm slices., The apoptotic cells on the slices were stained, using One Step TUNEL Apoptosis Assay Kit (C1090, Beyotime), according to the manufacturer's instructions. Image pro plus 6.0 software was used to calculate the number of TUNEL positive cells.

### Measurement of transepithelial resistance

To test intestinal epithelial barrier permeability, Caco-2 cells were seeded into upper transwell chambers. Using the Millicell-ERS system (Millipore, Burlington, MA, USA) to measure the Transepithelial electrical resistance (TEER).

### Statistical analyses

All statistical analyses were conducted using GraphPad Prism 8 software. Data are expressed as the means ± SDs. Correlation analysis in study were used Spearman's correlation analyses. The differences in quantitative data between the two groups were compared using the unpaired Student's scale t-test, Mann-Whitney U-tests or Dunnett's t-tests, as appropriate. When more than two groups of data were compared, one-way ANOVA was used. P values less than 0.05 were designated as significant differences.

## Results

### P2RY13 is upregulated in UC by Bioinformatics Analysis

**Figure 1:**
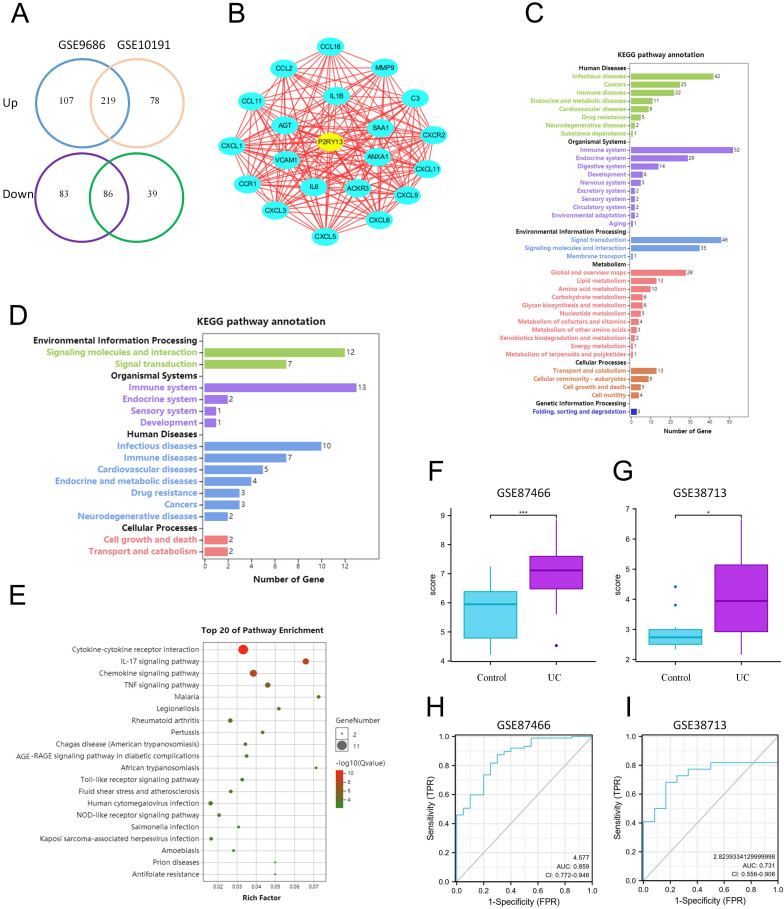
** P2RY13 is upregulated in UC by Bioinformatics Analysis. (A)** The DEGs were obtained from GSE9689 and GSE10191, 219 DEGs were upregulated and 86 DEGs were downregulated. **(B)** Cytoscape software (v3.8.0) was used to screen the core DEGs according to the analysis of DEGs. **(C)** KEGG enrichment analysis was performed for the DEGs from (A). **(D)** KEGG enrichment analysis was performed for 21 core DEGs. **(E)** Go enrichment analysis of core DEGs, was performed using Omicshare online tool. **(F-G)** Verification by two GEO datasets: GSE87466 and GSE38713. P2RY13 is upregulated in UC with significance. **(H-I)** ROC curve of the P2RY13 genes in UC in GSE87466 (AUC=0.859) and GSE38713 (AUC=0.731). AUC area under the ROC curve. KEGG, Kyoto Encyclopedia of Genes and Genomes; GO, Gene Ontology; DEGs, differentially expressed genes.

Two Gene Expression sequences (GSE9686 and GSE10191) of colonic mucosal tissues of UC patients and healthy controls from Gene Expression Omnibus (GEO) database were chosen for analysis. A total of 219 upregulated differentially expressed genes and 86 downregulated differentially expressed genes were obtained by using GEO2R online tools and Venn Diagram software (Figure 1A). Kyoto Encyclopedia of Genes and Genomes (KEGG) analysis found that these genes are involved in multiple signaling pathways and metabolism, regulating multiple systems, and participating in a variety of human diseases (Figure 1C). Cytoscape software (v3.8.0) was used to analyze the upregulated and downregulated differentially expressed genes. Twenty-one core differentially expressed genes, including P2RY13, were screened out (Figure 1B). The list of the 21 core differentially expressed genes were shown in Table S1. KEGG enrichment analysis found that these genes mainly affect signal transduction and signal molecule interaction, regulate the immune system, endocrine system and digestive system, and participate in the development of human infectious diseases, neoplastic growth, and immune diseases (Figure 1D). Gene Ontology (GO) enrichment analysis showed that 21 core differentially expressed were involved in cytokines-cytokine receptors interaction, IL-17 signaling pathway, chemokine signaling pathway, TNF signaling pathway, and other signaling pathways (Figure 1E). Among these 21 core differentially expressed genes, as a key inflammatory molecule, there are clear clues that P2RY13 may be associated with intestinal inflammation. To verify the accuracy of the expression of P2RY13, we selected two datasets GSE87466 and GSE38713. P2RY13 was significantly upregulated in both the two datasets (Figure 1F-G). Furthermore, we used these datasets to draw ROC curves and calculate area under the curve. Our results suggest that P2RY13 has a diagnostic value in UC (AUC = 0.859, AUC = 0.731) (Figure 1H-I). Bioinformatics results showed that the expression of P2RY13 was significantly increased in UC.

### P2RY13 expression was upregulated in colon tissue of patients with UC and correlated with the severity of UC

**Figure 2:**
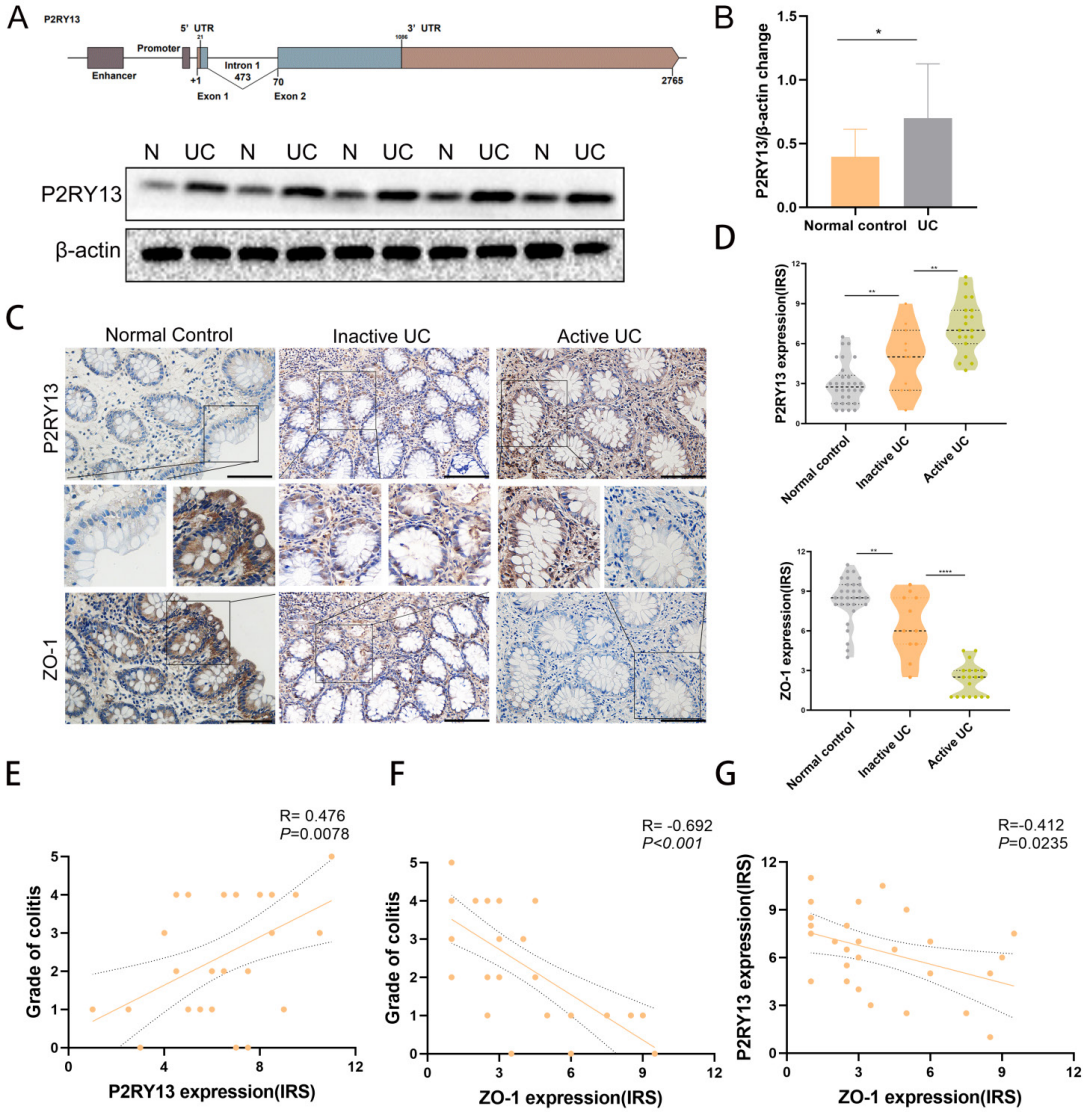
** P2RY13 expression was up-regulated in human ulcerative colitis (UC). (A)** Gene structure of P2RY13.Western blotting was performed to measure the levels of P2RY13 protein expression level in UC. N, normal colon; UC, ulcerative colitis. **(B)** Western blotting quantitative analysis of P2RY13 expression. n = 15. **(C)** Immunohistochemical staining of P2RY13 (×400) and ZO-1(×400). normal controls (n = 30), inactive UC (n = 11) and active UC (n = 19) tissues. Data for the scatter diagrams were generated from immunoreactive scores (IRSs). **(E)** The Spearmen's correlation between P2RY13 expression and colitis severity (r = 0.476, p=0.0078). **(F)** The Spearman's correlation between ZO-1 expression and colitis severity (r = -0.692, p<0.001). **(G)** The Spearman's correlation between P2RY13 and ZO-1 expression in epithelial cells of patients with UC (r=-0.412, p=0.0235). Data represent the means ± SEMs. *p < 0.05; **p < 0.01; ***p < 0.001; ****P < 0.0001.

To investigate whether P2RY13 expression is upregulated in colon tissue of UC patients. We obtained fresh specimens during colonoscopy. Gene structure of P2RY13 was shown (Figures 2A). Consistent with the bioinformatics analysis, Western blot showed that the expression of P2RY13 protein in inflamed mucosa of UC was higher than in normal tissues (Figures 2A and 2B). We used immunohistochemical (IHC) methods to probe for P2RY13 expression in colon tissue samples. P2RY13 was demonstrated in both cytoplasm and nucleus of epithelial cells. In addition, the expression of P2RY13 increased significantly in tissues from patients with UC, especially in UC flare-up (Figures 2C and 2D). Spearman's correlation analysis showed that the expression of P2RY13 in colon tissue was positively correlated with the severity of colitis (Figures 2E). To understand the relationship between P2RY13 and colitis, we examined the expression of tight junction protein ZO-1 in the same samples using immunohistochemistry. ZO-1 expression was low in UC tissues (Figure 2C-2F). Spearman's correlation analysis revealed that P2RY13 expression was negatively correlated with ZO-1 expression in UC tissues (Figure 2G). Thus, these data suggest that P2RY13 may promote the exacerbation of UC, and possibly mediates the intestinal epithelial barrier disruption in the development of UC in humans. The original Geboes Score (OGS) was used to assess the severity of colitis 17. Inactive UC includes patients in long-term remission with no endoscopic signs of colitis (OGS Grade 0 or 1). Active UC is diagnosed when acute signs of colitis are demonstrated in endoscopy (OGS score ≥ 2).

### Inhibition of P2RY13 activation attenuates DSS-induced colitis

**Figure 3:**
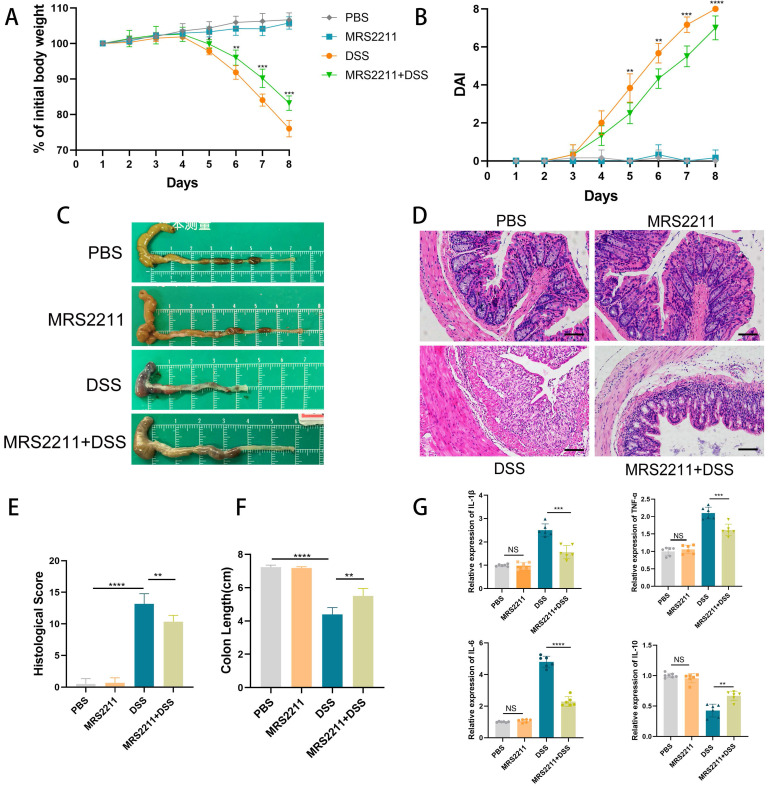
** Inactivation of P2RY13 attenuates DSS-induced colitis *in vivo*. (A-H)** 6-week-old WT mice (n=5-7) were treated with or without 4% DSS for 8 days and injected intraperitoneally with PBS or MRS2211 (1 mg/kg) daily from day 1 to day 7. Colitis induction was evaluated by body weight loss (A) expressed as a percentage of the initial body weight and by the DAI (B). Representative colon morphology and length of the mice are shown in (C) and are quantified in (F). Representative images of histological (×200) analyses are shown in (D) and are quantified in (E). (G) The mRNA expression of cytokines was measured in mouse colon tissues (n = 6 per group). Data represent the means ± SEMs. *p < 0.05; **p < 0.01; ***p < 0.001; ****P < 0.0001.

To further confirm that the promoting effect of P2RY13 on UC development, we next assessed the effect of P2RY13 pharmacological inhibition on the development of DSS-induced colitis. In our study, the mice treated with MRS2211 (specific inhibitor of P2RY13) exhibited a slower slower weight loss (Figure 3A), a lower Disease activity index (Figure 3B), significantly inhibited cecal edema and colon shortening (Figure 3C and 3F) and a lower histological score (Figure 3E), while MRS2211 alone has little influence on mice. MRS2211 treatment alleviated inflammation, epithelial damage and ulceration caused by DSS (Figure 3D). In addition, the mRNA expression of inflammatory cytokines IL-6, IL-1β and TNF-α was reduced in mice treated with MRS2211+ DSS, compared with mice treated with DSS, especially IL-6 expression, and the expression of mRNA encoding the IL-10 was increased (Figure 3G). These results demonstrate that activation of P2RY13 can exacerbate DSS-induced colitis.

### Inactivation of P2RY13 promotes recovery of intestinal mucosal barrier *in vivo* and vitro

**Figure 4:**
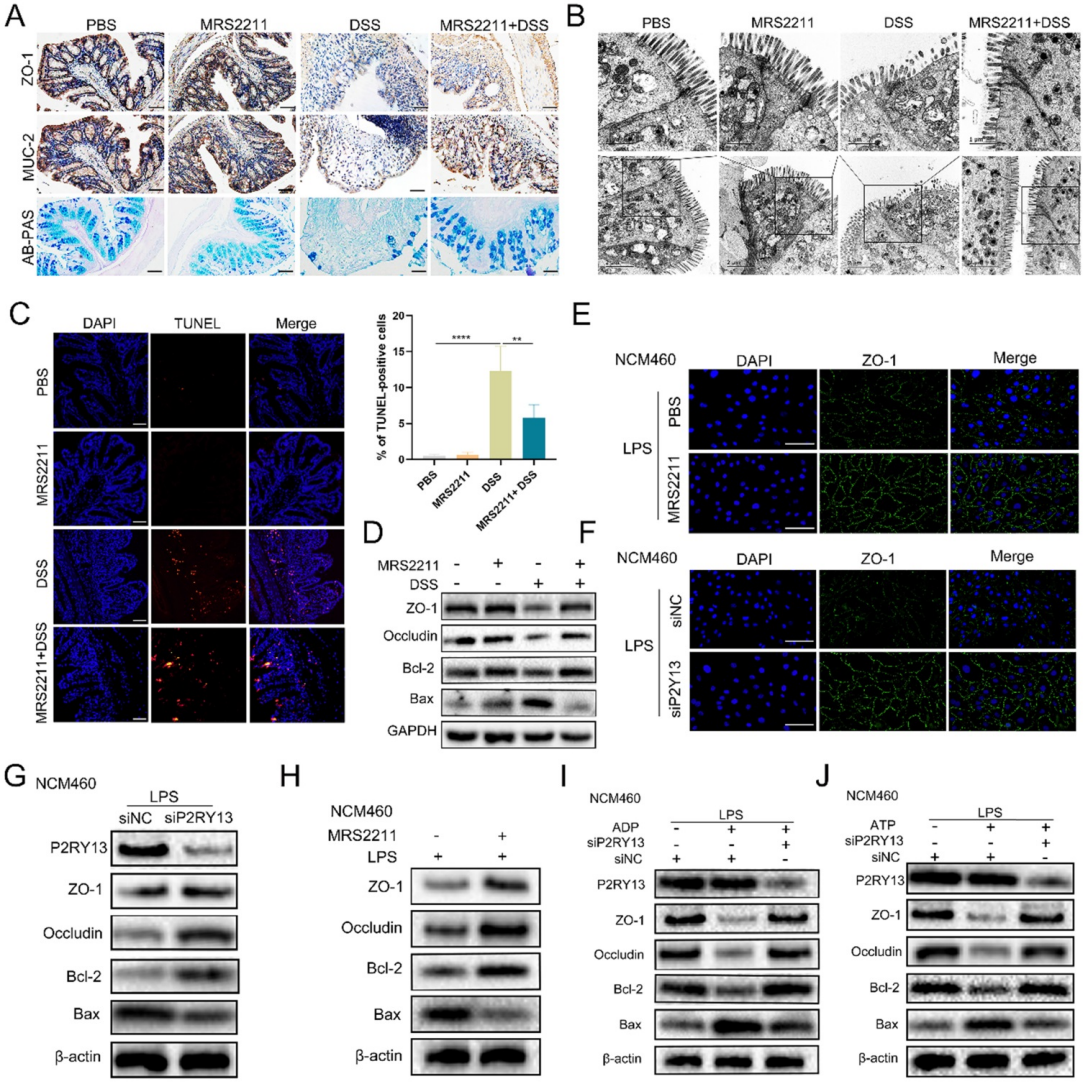
** Activation of P2RY13 can aggravate intestinal mucosal barrier destruction *in vivo* and vitro. (A-D)** Representative images of AB-PAS (×200) and immunohistochemical (×200) of ZO-1 and MUC-2 are shown in (A). Electron micrograph images of colonic segments harvested from mice are shown in (B). Upper scale bar =1 µm. Bottom scale bar = 2 um. TUNEL (×200) staining of colon tissue and Statistical analysis of TUNEL-positive cells are shown in (C). (D) Western blotting was performed to measure the levels of ZO-1, Occludin, Bcl-2 and Bax in colon tissues. Groups are identified as shown. **(E-H)** NCM460 cells were preincubated with 10 uM of MRS2211 for 1h before being treated with LPS (1 ug/ml) for 24 h. At 24hr after the stimulation of LPS, the immunostained (× 400) with ZO-1 (green) and DAPI (blue) are shown in (E). Western blotting was performed to measure the levels of ZO-1, Occludin, Bcl-2 and Bax are shown in (H). NCM460 cells transfected with P2RY13-targeting siRNA (siP2RY13-2) or nontargeting siRNAs (siNC) and stimulated with LPS, the immunostained (× 400) with ZO-1 (green) and DAPI (blue) are shown in (F). Western blotting was performed to measure the levels of P2RY13, ZO-1, Occludin, Bcl-2 and Bax are shown in (G). **(I-J)** LPS-primed NCM460 cells transfected with P2RY13-targeting siRNA (siP2RY13-2) or nontargeting siRNAs (siNC) were stimulated with ADP (100 µM) (I) or ATP (100 µM) (J) for 2 h, Western blotting was performed to measure the levels of P2RY13, ZO-1, Occludin, Bcl-2 and Bax. Data represent the means ± SEMs. *p < 0.05; **p < 0.01; ***p < 0.001; ****P < 0.0001.

Previous studies demonstrated that activation of P2RY13 in mouse pancreatic insulinoma MIN6C4 cells had a pro-apoptotic effect 18, and that the loss of P2RY13 reduced myenteric neuron death in the enteric nervous system of mice induced by high-fat diet 19. In our study, we found that P2RY13 expression was negatively correlated with ZO-1 expression in UC. To determine the role of P2RY13 in the pathogenesis of IBD, we examined the indicators of intestinal mucosal barrier disruption in mouse intestinal tissue. In DSS-induced colitis, treatment with MRS2211 significantly increased the expression of ZO-1 and MUC-2 (Figure 4A). The results of AB-PAS staining demonstrated an increase of mucin secretion from colonic glands when P2RY13 activity was inhibited with MRS2211 (Figure 4A). The changes in tight junctions due to DSS were improved with MRS2211 treatment (Figure 4B). MRS2211 treatment significantly decreased the number of TUNEL positive cells (Figure 4C). Moreover, the results of Western blotting showed that colon tissues from the mice treated with MRS2211 exhibited higher levels of ZO-1, occludin and Bcl-2, and lower levels of Bax than in DSS-treated mice (Figure 4D). These results indicated that P2RY13 pharmacological inhibition can alleviate DSS-induced colitis by reducing the destruction of intestinal mucosal barrier.

To determine the role of P2RY13 in the pathogenesis of IBD, we pretreated NCM460 cells with MRS2211 and we also used the NCM460 cells was transfected with P2RY13-targeting siRNA (siP2RY13) or nontargeting siRNAs (siNC). Knockout of P2RY13 or pretreatment with MRS2211 suppressed the LPS-induced reduce in expression of ZO-1 detected by immunofluorescence (Figure 4E and 4F). Consistent with this finding, the Western blotting results showed that the LPS-mediated decreases in the levels of ZO-1, occludin, Bcl-2 were upregulated and that Bax expression was reduced in the MRS2211-pretreated NCM460 cells or the siP2RY13-transfected NCM460 cells (Figure 4G and 4H). To further understand the effect of P2RY13 on intestinal epithelial barrier function, we cultured Caco-2 monolayer cells to measure transepithelial electrical resistance (TEER). Pretreatment with MRS2211 significantly alleviated the reduction of TEER induced by LPS (Figure S1A). Our data showed that inhibition of P2RY13 reduced LPS-induced disruption of tight junctions of intestinal epithelium, cell apoptosis and destruction of the intestinal barrier. P2RY13 can be activated by both ATP and ADP. We also confirmed that ADP- and ATP-promoted disruption of tight junctions of intestinal epithelium and apoptosis are primarily mediated by P2RY13 in NCM460 cells (Figure 4I and 4J).

Collectively, these results suggest that the activation of P2RY13 can aggravate the destruction of intestinal mucosal barrier.

### P2RY13 promotes UC by activating the IL-6/STAT3 signaling pathway

In IBD patients and some animal models with colitis, there are high levels of IL-6 expression and high levels of STAT3 phosphorylation, which are related to disease activity 20. IL-6/STAT3 pathway plays a crucial role in the development of DSS-induced colitis 21. We have confirmed that the activation of P2RY13 can aggravate UC. Subsequently, our aim is to elucidate the molecular mechanism of P2RY13 involvement in UC. Increasing studies have shown that inhibition of P2RY13 activity by MRS2211 can inactivate IL-6/STAT3 pathway 16, 22. In our study, bioinformatics analyses suggested an association between P2RY13 and IL-6 levels in UC. In addition, our results show that MRS2211 treatment can significantly inhibit the expression of IL-6 in DSS-induced colitis. This led us to speculate whether P2RY13 aggravates UC by activating IL-6/STAT3 pathway.

**Figure 5:**
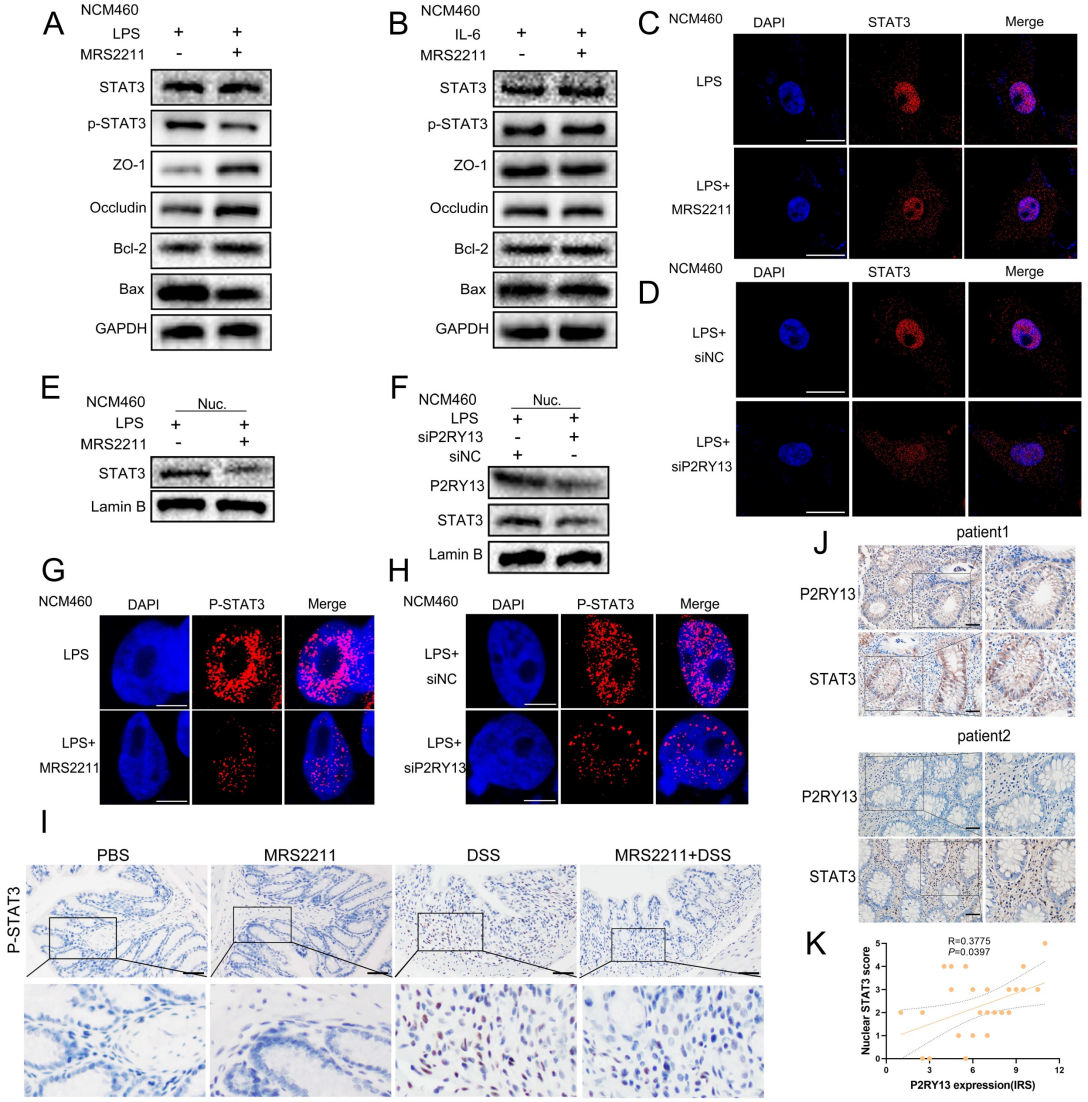
** P2RY13 promotes IL-6-mediated STAT3 phosphorylation and nuclear translocation. (A)** NCM460 cells were preincubated with 10 uM of MRS2211 for 1h before being treated with LPS (1 ug/ml) for 24 h. At 24h after the stimulation of LPS, Western blotting was performed to measure the levels of STAT3, p-STAT3, ZO-1, Occludin, Bcl-2 and Bax. **(B)** NCM460 cells were preincubated with 10 uM of MRS2211 for 1 h before being treated with IL-6 (20 ng/ml) for 12 h. At 12hr after the stimulation of IL-6, Western blotting was performed to measure the levels of STAT3, p-STAT3, ZO-1, Occludin, Bcl-2 and Bax. **(C)** Confocal microscopy showing changes in subcellular localization of STAT3 in the NCM460 cells were preincubated with 10 uM of MRS2211 before being treated with LPS. The immunostained (× 600) with STAT3 (red) and DAPI (blue). **(D)** NCM460 cells transfected with P2RY13-targeting siRNA (siP2RY13-2) or nontargeting siRNAs (siNC) and stimulated with LPS, Confocal microscopy showing changes in subcellular localization of STAT3.The immunostained (× 600) with STAT3 (red) and DAPI (blue). **(E)** NCM460 cells were preincubated with 10 uM of MRS2211 before being treated with LPS, Western blotting was performed to measure the levels of STAT3 of nucleus. **(F)** NCM460 cells transfected with P2RY13-targeting siRNA (siP2RY13-2) or nontargeting siRNAs (siNC) and stimulate with LPS, Western blotting was performed to measure the levels of STAT3, P2RY13 of nucleus. **(G)** Confocal microscopy showing the expression of P-STAT3 in the NCM460 cells were preincubated with 10 uM of MRS2211 before being treated with LPS. The immunostained with P-STAT3 (red) and DAPI (blue). **(H)** NCM460 cells transfected with P2RY13-targeting siRNA (siP2RY13-2) or nontargeting siRNAs (siNC) and stimulated with LPS, Confocal microscopy showing the expression of P-STAT3.The immunostained with P-STAT3 (red) and DAPI (blue). **(I)** Immunohistochemical (× 200) of the p-STAT3 protein in colonic tissues from WT mice were treated with or without 4% DSS for 8 days and injected intraperitoneally with PBS or MRS2211 (1 mg/kg) daily from day 1 to day 7. **(J)** Representative micrographs of immunohistochemical staining (× 200) of STAT3 and P2RY13 in matched specimens of human ulcerative colitis. Insets show staining of specimens in more detail. **(K)** Statistical analysis of Spearman's correlation between immunohistochemistry staining scores of P2RY13 expression and STAT3 nuclear localization. P2RY13 staining was scored according to immune response scores (IRSs). STAT3 staining was quantified by nuclear localization as scores of 0 (<10%), 1 (10%-20%), 2 (20%-40%), 3 (40%-60%), 4 (60%-80%) or 5 (>80%). Data represent the means ± SEMs. *p < 0.05; **p < 0.01; ***p < 0.001; ****P < 0.0001.

We hypothesized that MRS2211 treatment suppresses the activation of the IL-6/STAT3 pathway. As the hypothesis, knockout of P2RY13 or pretreatment with MRS2211 suppresses the LPS-induced increase in the mRNA levels of IL-6(Figure S1B and C). In the LPS-primed NCM460 cells, MRS2211 treatment significantly inhibited the expression of P-STAT3 (Figure 5A and 5G). However, in the IL-6-primed NCM460 cells, MRS2211 treatment had no significant effect on the expressions of P-STAT3, ZO-1, occludin, Bcl-2 and Bax (Figure 5B), suggesting that the activation of STAT3 by P2RY13 is mediated by IL-6. In addition to inhibiting P2RY13, MRS2211 also can inhibit P2Y1 or P2Y12 at higher concentrations 11. To eliminate the role of these two receptors in MRS2211 on the IL-6/STAT3 pathway, we treated LPS-primed NCM460 cells with MRS2179 (an P2RY1 antagonist) or ticagrelor (an P2RY12 antagonist). The Western blotting results showed that there was no significant change in the expression of P-STAT3 (Figure S2 A and B). We further examined the effects of P2RY1 and P2RY12 inhibition on IL-6 expression, inhibition of P2RY1 and P2RY12 had no significant change in the mRNA levels of IL-6 (Figure S2C and D). The results showed that P2RY1 and P2RY12 had no effect on the expression of IL-6 and P-STAT3. Knockout of P2RY13 also significantly inhibited the expression of P-STAT3 (Figure 5H). These results suggest that P2RY13 activation can promote STAT3 phosphorylation. Meanwhile, pharmacological inhibition of P2RY13 with MRS2211 significantly abrogated LPS-induced translocation of STAT3 from the cytoplasm to the nucleus (Figure 5C, 5E and Figure S1D). The same phenomenon was observed in P2RY13 knockdown cells (Figure 5D, 5F and Figure S1E). In mice, MRS2211 treatment similarly inhibited the phosphorylation of STAT3 in DSS-induced colitis (Figure 5I). High P2RY13 protein levels confirmed in colon tissue of UC through immunohistochemistry showed significant correlation with nuclear STAT3 staining (Figure 5J and 5K).

Collectively, these observations imply that P2RY13 may promote UC by activating the IL-6/STAT3 signaling pathway.

### Loss of STAT3 can reverse the aggravation of DSS-induced colitis caused by P2RY13 activation

**Figure 6:**
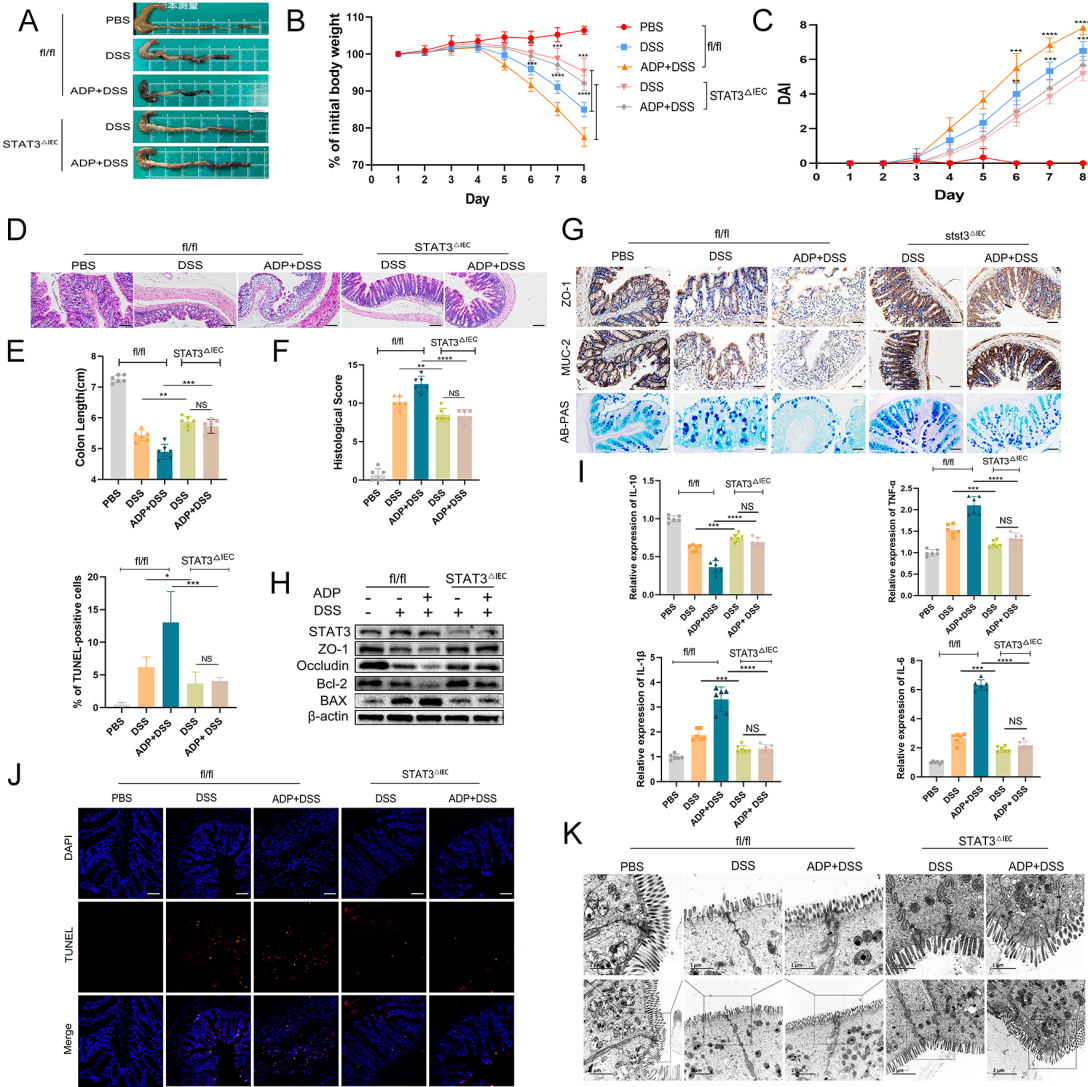
** P2RY13 can aggravate DSS-induced colitis by STAT3. (A-K)** Both fl/fl and STAT3^△IEC^ mice (n = 5-7 per group) were treated with or without 2.5% DSS for 8 days and received PBS or ADP (100 mg/kg) daily by oral gavage from day 1 to day 7. Representative colon morphology and length are shown in (A) and are quantified in (E). Mouse body weight (B) and disease activity index (DAI) (C) were recorded daily. Representative images of histological analyses are shown in (D) and are quantified in (F). Representative images of AB-PAS (×200) and immunohistochemical (×200) of ZO-1 and MUC-2 are shown in (G). Western blotting was performed to measure the levels of STAT3, ZO-1, Occludin, Bcl-2 and Bax are shown in (H). The mRNA expression of cytokines was measured in mouse colon tissues are shown in (I). TUNEL (× 200) staining of colon tissue and Statistical analysis of TUNEL-positive cells are shown in (J). Electron micrograph images of colonic segments harvested from mice are shown in (K) upper Scale bar =1 µm. bottom Scale bar = 2 um. Data are mean ± SEM, *p < 0.05; **p < 0.01; ***p < 0.001; ****P < 0.0001.

To further verify our hypothesis that activation of P2RY13 may promote the development of colitis through the IL-6/STAT3 signaling pathway, we generated intestinal epithelial cells STAT3 conditional knockout (STAT3^△IEC^) mouse strain as described in the Materials and Methods. Genotypes were shown (Figure S3). P2RY13 is more sensitive to ADP than ATP. In this study, we selected ADP as the agonist of P2RY13. Both STAT3^△IEC^ mice and fl/fl mice were treated with ADP and DSS. In the DSS + ADP -induced colitis model, STAT3^△IEC^ mice exhibited a longer colon size ratio (Figure 6A and 6E), a slower weight loss (Figure 6B), a lower histological score (Figure 6F), a lower disease activity index (DAI) (Figure 6C), and milder tissue damage, ulceration, and inflammation of histological analysis (Figure 6D) than fl/fl mice. Compared to fl/fl mice, the STAT3^△IEC^ mice had increased expressions of ZO-1 and MUC-2 (Figure 6G), mucin secretion from colonic glands (Figure 6G), levels of ZO-1, occluding, and Bcl-2 by Western blotting (Figure 6H), and had decreased levels of Bax (Figure 6H). Furthermore, STAT3^△IEC^ mice suppressed inflammatory cytokine expression (IL-6, IL-1β, and TNF-α) and increased the expression of IL-10 (Figure 6I). By transmission electron microscopy, STAT3^△IEC^ mice improved the changes in tight junctions (Figure 6K).

Taken together, these results indicate IL-6/STAT3 pathway plays an important role in the regulation of P2RY13-aggravated intestinal inflammation.

## Discussion

Genome-wide association studies (GWAS) has identified many gene loci involved in the development of UC 23. Although the pathogenesis of UC remains unclear, genetic factors certainly play an important role in the onset and development of UC. In order to find the gene loci related to the development of UC, we select the datasets GSE9686 and GSE10191, which were obtained from UC patients and healthy controls. We focused on 21 core differential genes. Among them, P2RY13, a purinergic receptor, which can be activated by ATP or ADP, attracted our attention, apart from the gene that has been shown to play a role in UC.

Intracellular ATP has long been considered as an energy carrier. In recent years, it has been found that intact cells can release some cellular ATP 24, which has been shown to be related to inflammation and immunity 25. The purinergic signaling hypothesis was proposed in 1972, with purinergic receptors among the most widely studied. Purinergic receptors are divided into three main families: P2X receptors, P2Y receptors, and P1 receptors. The P2Y receptor is a G protein-coupled receptor (GPCRs) that can recognize ATP, ADP, UTP, UDP and UDP-glucose. Different subtypes of P2Y receptors mediate many pathophysiological processes, including apoptosis, immune regulation, cell proliferation and differentiation 14. Increasing evidence show that P2Y receptor is involved in the pathogenesis of inflammatory diseases and tumors 14. While the P2Y1, P2Y2 and P2Y6 of P2Y receptor involved in the development of UC has been described 12, 26-27, the role of P2RY13 in UC has never been investigated. P2RY13 is one of the important members of the P2Y receptor family and is synthesized by 354 amino acid codes. Studies have shown that P2RY13 exerts biological effects through conjugation with G_i_ protein to inhibit adenylate cyclase activation and cyclic adenosine monophosphate synthesis 15. In recent decades, more evidence show that P2RY13 receptors are involved in the pathogenesis of several human diseases, such as asthma, viral infections, nervous system diseases, diabetes, liver disease, and bone dysplasia. P2RY13 receptor mediates many pathophysiological processes, such as apoptosis, autophagy, proliferation and metabolism 11, 16, 28-29.

In GSE10191 and GSE9686 datasets, P2RY13 expression was upregulated in UC. It is reasonable to speculate that P2RY13 may play a role in UC. To verify the accuracy of the results and the expression of P2RY13, we selected two datasets GSE87466 and GSE38713. P2RY13 was significantly upregulated in both datasets. Meanwhile, we use these two datasets to draw ROC curves and calculate area under the curve. The results suggested that P2RY13 have higher diagnostic value in UC. According to the above results, we selected P2RY13 as the focus of the following study.

To determine whether P2RY13 is highly expressed in UC, we investigated the expression of P2RY13 in the inflamed intestinal tissue of UC patients. We found that P2RY13 was highly expressed in the inflamed intestinal tissues of UC patients. In particular, our results showed that P2RY13 was positively correlated with disease activity of UC and negatively correlated with the expression of intestinal barrier related proteins of UC. To investigate the influence of P2RY13 on the development of UC, we first chose to explore the role of P2RY13 in the mouse model of DSS induced colitis. P2RY13 can be activated by ADP or ATP, but is highly sensitive to ADP. Studies have shown that in DSS-induced colitis, the release of ADP and ATP from colon tissue was increased, and that ADP can aggravate DSS-induced colitis 12. To explore whether P2RY13 activated by ADP or ATP has an impact on colitis, we used MRS2211 to inhibit the activity of P2RY13 in the DSS-induced colitis. Interestingly, we found that MRS2211 treatment reduced colon shortening and alleviated weight loss and tissue damage caused by DSS. The results were consistent with the results of Bioinformatics Analysis and intestinal tissue examination in UC patients. Furthermore, it was accompanied by reduction of proinflammatory factors in colonic tissue.

A normal intestinal barrier prevents luminal antigens, microorganisms and toxins from entering the body milieu, and maintains mucosal immune homeostasis to prevent uncontrolled inflammation 30. Disruption of the intestinal barrier can be caused by a variety of factors, such as epithelial-specific congenital immune deficiency, increased epithelial permeability, disruption of epithelial cell integrity, decreased mucus production, and increased production of pro-inflammatory factors 30-31. Increasing studies suggest that the disruption of the intestinal mucosal barrier is the etiology of IBD 32. In our study, IHC results showed that P2RY13 expression was negatively correlated with ZO-1 expression in UC tissues. Many studies have shown that the activation of P2RY13 can promote apoptosis 18-19 and increase the release of pro-inflammatory factors 11, 16, 22. Damage to colon cells and release of pro-inflammatory factors can damage the integrity of intestinal epithelium and further aggravate the destruction of the intestinal barrier. Therefore, we speculated whether MRS2211 alleviates colitis by reducing the destruction of the intestinal mucosal barrier. To test this hypothesis, we examined several indicators related to the intestinal mucosal barrier. In our results, inhibition of P2RY13 activity significantly increased the expression of intestinal barrier related proteins and the mucin secretion from colonic glands *in vivo*. Through transmission electron microscopy, we observed that MRS2211 treatment can restore the destruction of intestinal tight junction. Meanwhile, we observed a decrease in the number of TUNEL positive cells after MRS2211 treatment, suggesting that the activation of P2RY13 can promote colon cell apoptosis. This is consistent with LPS-induced NCM460 cell model, where knockdown or pharmacological inhibition of P2RY13 increased the expression of intestinal tight junction protein and reduced apoptosis. By detecting transepithelial electrical resistance of Caco-2 monolayer cells, pharmacological inhibition of P2RY13 significantly alleviated LPS-induced TEER reduction. We found that intestinal barrier destruction induced by ADP and ATP were mediated by P2RY13 activation *in vitro*. Our data strongly implicate a role of P2RY13 activation in the development of colitis. Future studies will require intestinal epithelial cell-specific knockout P2RY13 mice to definitively implicate IECs (or other cell types) *in vivo*.

We then investigated the mechanism involved in the destruction of intestinal mucosal barrier of P2RY13. By examining the mucosal expression of inflammatory cytokines in mice, we found MRS2211 treatment significantly inhibited the expression of IL-6. In addition, results of bioinformatics analysis showed that P2RY13 was correlated with IL-6. Some studies have confirmed that P2RY13 activation can increase IL-6 expression and affect STAT3 16, 22. The levels of IL-6 in colonic mucosa and serum of patients with active IBD were higher than the patients with inactive disease or normal controls, IL-6 mRNA levels were highest in patients with active IBD 21. As an important downstream target of IL-6, STAT3 has a high level of phosphorylation in IBD patients and in some animal models of colitis or enteritis 21, 33. STAT3 phosphorylation was positively correlated with disease activity 33. The activation of IL-6/STAT3 pathway is involved in the occurrence and development of colitis. Inhibition of IL-6 or its receptor has shown promising prospects in the treatment of IBD 34. This led us to question whether the effect of P2RY13 on UC is mediated by the IL-6/STAT3 pathway. We first verified the effect of P2RY13 on IL-6 expression *in vitro* that blocking P2RY13 activation and knockout P2RY13 inhibited the production of IL-6 in inflammatory stimulated NCM460 cell. STAT3 protein is a potential cytoplasmic transcription factor. After IL-6 stimulation, STAT3 can be phosphorylated, dimerized, and transferred to the nucleus to induce transcription 33. To investigate whether P2RY13 can affect STAT3 activation, we detected the phosphorylated STAT3 expression and STAT3 expression levels in and out of the nucleus. The results indicate that STAT3 phosphorylation and nuclear translocation was attenuated by treatment with the P2RY13 antagonist MRS2211 in inflammatory stimulated NCM460 cell and DSS induced colitis. This was consistent with our hypothesis, in IL-6-induced NCM460 cells, the above indices did not change, suggesting that the activation of STAT3 by P2RY13 is mediated by IL-6. MRS2211 can also inhibit P2Y1 and P2Y12, but the selectivity is very low compared with P2RY13. Importantly, P2Y1 and P2Y12 had no effect on STAT3 phosphorylation and the expression of IL-6. The effect of MRS2211 on STAT3 is produced by inhibiting P2RY13. To further implicate P2RY13, we transfected NCM460 cells with P2RY13-targeting siRNA (siP2RY13) and confirmed that P2RY13 contributes to STAT3 phosphorylation and nuclear translocation. We confirmed that high P2RY13 protein levels showed significant correlation with nuclear STAT3 staining in UC.

To directly assess the contribution of STAT3 in the P2RY13-induced exacerbation of colitis,we used the Flox-villin-Cre system to delete STAT3 from intestinal epithelium of mice. STAT3^△IEC^ mice were modestly protected after ADP treatment, as demonstrated by heavier body weight, lighter disease activity index score, longer intestinal length, lower histological score, reduced proinflammatory factor expression, reduced intestinal tight junction destruction, and reduced colonic cell injury. Our results suggest that the absence of STAT3 would attenuate the aggravation of intestinal mucosal barrier disruption caused by P2RY13 activation.

In conclusion, we provide evidence that P2RY13 promotes the development of UC by damaging the intestinal mucosal barrier via IL-6/STAT3 pathway activation. P2RY13 may be an attractive therapeutic target for UC.

## Supplementary Material

Supplementary figures and tables.

## Figures and Tables

**Table 1:** Patient characteristics

Characteristic	Normal control (n = 30)	Inactive UC (n = 11)	Active UC (n = 19)
**Gender**			
Male	14	7	9
Female	16	4	10
Age (years ± SD)	45.8 ± 13.5	43.1 ± 13.58	48.47 ± 12.82
**Disease duration (years ± SD)**	-	3.42 ± 1.27	2.24 ± 2.47
Extent of disease			
Extensive colitis	-	3	12
Left-sided colitis	-	5	5
Proctitis	-	3	2
**Treatment**			
Amino salicylates	-	3	10
Immune modulator use	-	0	1
Corticosteroids	-	5	3
None	-	3	5
**Pathological severity**			
Grade 0-1	-	11	-
Grade 2-5	-	-	19

## Abbreviations

ZO-1: Zona occludens 1
MUC-2: mucin 2
P2RY13: P2Y purinoceptor 13
ATP: adenosine triphosphate
DSS: dextran sulphate sodium
HE: hematoxylin and eosin
IBD: inflammatory bowel disease
TNF: tumor necrosis factor
UC: ulcerative colitis
DAI: disease activity inde
ADP: Adenosine diphosphate
STAT3: signal transducer and activator of transcription 3
UTP: Uridine 5'-triphosphate
UDP: Uridine diphosphate
LPS: Lipopolysaccharide
NLRP3: NACHT, LRR, and PYD domains-containing protein 3
