# Supplementary material for: P2RY13 Exacerbates Intestinal Inflammation by Damaging the Intestinal Mucosal Barrier via Activating IL-6/STAT3 Pathway
Source: Int J Biol Sci. 2022 Aug 1;18(13):5056–69. doi: 10.7150/ijbs.74304 (PMC9379400; doi:10.7150/ijbs.74304)
Supplement: Supplementary file 1 — Supplementary figures and tables. [file ijbsv18p5056s1.pdf]

Table S1.

Gene bank accession	Genes	Cluster	Values
NM_023914	P2RY13	Clustered	13
NM_002993	CXCL6	Clustered	13.26797
NM_000029	AGT	Clustered	12.06536
NM_000700	ANXA1	Clustered	13
NM_000576	IL1B	Clustered	12.42647
NM_005409	CXCL11	Clustered	13.26797
NM_002988	CCL18	Clustered	12.87619
NM_080682	VCAM1	Clustered	13.26797
NM_001295	CCR1	Clustered	13.26797
NM_199161	SAA1	Seed	13.76667
NM_001511	CXCL1	Clustered	13.26797
NM_001557	CXCR2	Clustered	13.26797
NM_002416	CXCL9	Clustered	13.26797
NM_002982	CCL2	Clustered	13.76667
NM_000600	IL6	Clustered	13.26797
NM_000064	C3	Clustered	12.15033
NM_002986	CCL11	Clustered	13
NM_004994	MMP9	Clustered	13.48529
NM_020311	ACKR3	Clustered	13
NM_002994	CXCL5	Clustered	13.26797
NM_002090	CXCL3	Clustered	13.26797

Table S2. List of primers used

Target		Sequence 5’-3’	Purpose
m-IL-1β	Forward	5’-GGGCCTCAAAGGAAAGAATCT-3’	qPCR
	Reverse	5’-GAGGTGCTGATGTACCAGTTGG-3’	
m-IL-6	Forward	5’-CTGGGAAATCGTGGAAATGAG-3’	qPCR
	Reverse	5’-AAGGACTCTGGCTTTGTCTTTCT-3’	
m-TNF-α	Forward	5’-TCCCCAAAGGGATGAGAAGTT-3’	qPCR
	Reverse	5’-GAGGAGGTTGACTTTCTCCTGG-3’	
m-IL-10	Forward	5’-TACAGCCGGGAAGACAATAACT-3’	qPCR
	Reverse	5’-AGGAGTCGGTTAGCAGTATGTTG-3’	
m-GAPDH	Forward	5’- TGCTGTCCCTGTATGCCTCTG-3’	qPCR
	Reverse	5’- TTGATGTCACGCACGATTTCC-3’	

h-IL-6	Forward	5'-TCAGCCCTGAGAAAGGAGACAT-3'	qPCR
	Reverse	5'-GCTCTGGCTTGTTCCTCACTACT-3'	
h-GAPDH	Forward	5'- ACAACTTTGGTATCGTGGAAGG-3'	qPCR
	Reverse	5'- GCCATCACGCCACAGTTTC-3'	
h-P2RY13	Forward	5'-TTTTGTTCTTCATCTCCCTGCC-3'	qPCR
	Reverse	5'-ACAAAGAAGACAGCCACGACAA-3'	

Table S3 primer for Mouse genotypes

Target		Sequence 5'-3'
STAT3 flox	Forward	5'-TTGACCTGTGCTCCTACAAAAA-3'
	Reverse	5'-CCCTAGATTAGGCCAGCACA-3'
Villin Cre	Forward	5'-ATTTGCCTGCATTACCGGTC-3'
	Reverse	5'-ATCAACGTTTTCTTTTCGG-3'

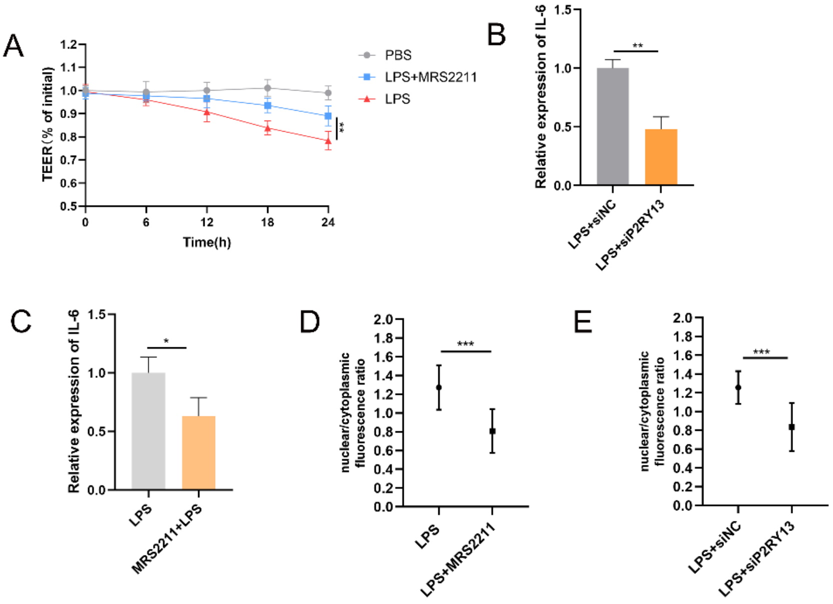

Figure S1.(A) Caco-2 monolayer cells were preincubated with 10 uM of MRS2211 for 1h before being treated with LPS (1 ug/ml) for 24 h.Transepithelial electrical resistance (TEER) was measured. (B)NCM460 cells transfected with P2RY13-targeting siRNA (siP2RY13-2) or nontargeting siRNAs (siNC) and stimulated with LPS for 12 h. At 12h after the stimulation of LPS, the mRNA expression of IL-6 was detected . (C) NCM460 cells were preincubated with 10 uM of MRS2211 for 1h before being treated with LPS (1 ug/ml) for 12 h. At 12h after the stimulation of LPS, the mRNA expression of IL-6 was detected. (D) Ratio of nuclear to cytoplasmic fluorescencen of STAT3 in Figure5 C was assessed using the Image J. (n=10).(E) Ratio of nuclear to cytoplasmic fluorescencen of STAT3 in Figure5 D was assessed using the

Image J. (n=10). Data are mean ± SEM, *p < 0.05; **p < 0.01; ***p < 0.001; ****P < 0.0001.

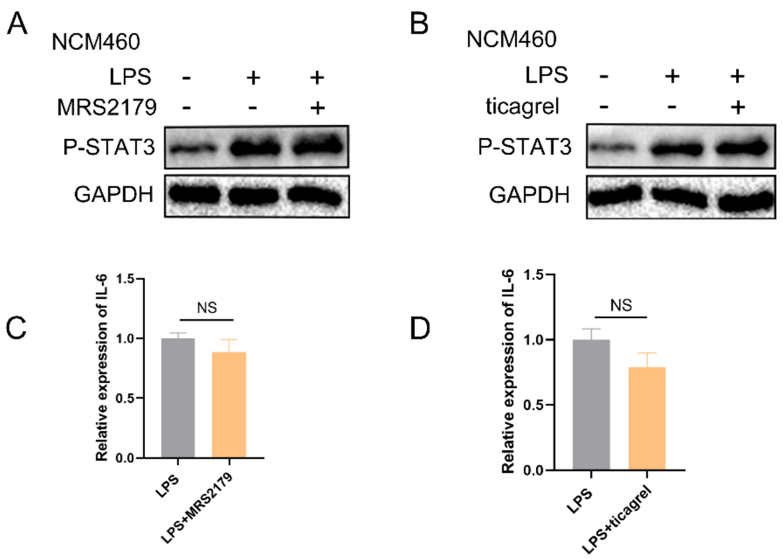

Figure S2. (A) NCM460 cells were preincubated with 10 uM of MRS2179 for 1h before being treated with LPS (1 ug/ml) for 24 h. At 24hr after the stimulation of LPS, Western blotting was performed to measure the levels of p-STAT3. (B) NCM460 cells were preincubated with 0.1 uM of ticagrelor for 1h before being treated with LPS (1 ug/ml) for 24 h. At 24hr after the stimulation of LPS, Western blotting was performed to measure the levels of p-STAT3. (C) NCM460 cells were preincubated with 10 uM of MRS2179 for 1h before being treated with LPS (1 ug/ml) for 12 h. At 12hr after the stimulation of LPS, the mRNA expression of IL-6 was detected . (D) NCM460 cells were preincubated with 0.1 uM of ticagrelor for 1h before being treated with LPS (1 ug/ml) for 12 h. At 12hr after the stimulation of LPS, the mRNA expression of IL-6 was detected .

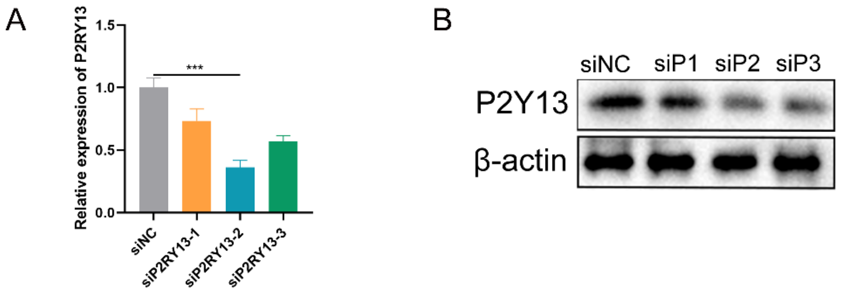

Figure S3. NCM460 cells transfected with nontargeting siRNAs (siNC),P2RY13-targeting siRNA (siP2RY13-1) ,P2RY13-targeting siRNA (siP2RY13-2) and P2RY13-targeting siRNA (siP2RY13-3). (A) the mRNA expression of P2RY13 was detected. (B)Western blotting was performed to

measure the levels of P2RY13.siP1, siP2RY13-1; siP2, siP2RY13-2; siP3, siP2RY13-3;

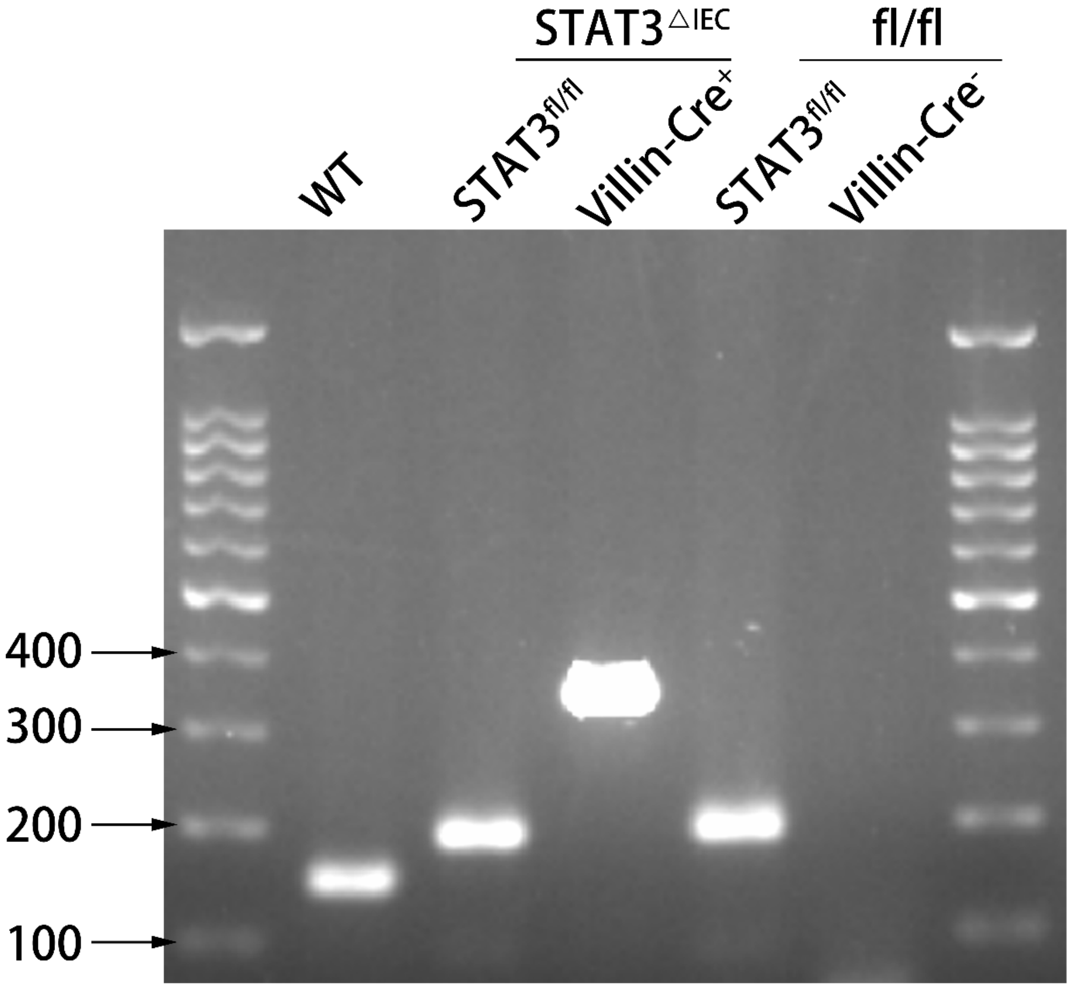

Figure S4. Representative PCR images from WT, STAT3$^{\triangle IEC}$ and STAT3$^{fl/fl}$ mice.
